# Colossal negative thermal expansion in reduced layered ruthenate

**DOI:** 10.1038/ncomms14102

**Published:** 2017-01-10

**Authors:** Koshi Takenaka, Yoshihiko Okamoto, Tsubasa Shinoda, Naoyuki Katayama, Yuki Sakai

**Affiliations:** 1 Department of Applied Physics, Nagoya University, Furo-cho, Chikusa-ku, Nagoya 464-8603, Japan; 2 Institute for Advanced Research, Nagoya University, Furo-cho, Chikusa-ku, Nagoya 464-8601, Japan; 3 Kanagawa Academy of Science and Technology, KSP, 3-2-1 Sakado, Takatsu-ku, Kawasaki 213-0012, Japan

## Abstract

Large negative thermal expansion (NTE) has been discovered during the last decade in materials of various kinds, particularly materials associated with a magnetic, ferroelectric or charge-transfer phase transition. Such NTE materials have attracted considerable attention for use as thermal-expansion compensators. Here, we report the discovery of giant NTE for reduced layered ruthenate. The total volume change related to NTE reaches 6.7% in dilatometry, a value twice as large as the largest volume change reported to date. We observed a giant negative coefficient of linear thermal expansion *α*=−115 × 10^−6^ K^−1^ over 200 K interval below 345 K. This dilatometric NTE is too large to be attributable to the crystallographic unit-cell volume variation with temperature. The highly anisotropic thermal expansion of the crystal grains might underlie giant bulk NTE via microstructural effects consuming open spaces in the sintered body on heating.

## Abstract (web summary)

Materials displaying negative thermal expansion have received interest as thermal-expansion compensators. Here, authors report very large total volume change associated with this effect in reduced calcium ruthenates and propose a microstructural origin driven by highly anisotropic thermal lattice expansion.

Rapid advances in modern technology require industrial materials that can adapt to severe conditions. One means of adaptation is controlled thermal expansion. Although thermal expansion is typically a 10^−5^ to 10^−6^ change in length, even such a minute change fatally degrades the performance of devices and instruments in many fields of industry. Consequently, negative thermal expansion (NTE) materials^1^,^2^,^3^,^4^,^5^, materials that contract during heating, have been attracting great attention because NTE materials can be used to tune the overall thermal expansion of materials. Remarkable development in the field of NTE materials was provoked by the discovery of large, isotropic NTE over a wide range of temperatures *T* in ZrW_2_O_8_ (ref. 6). This NTE originates from a characteristic crystal structure called a flexible network. This class of NTE materials reaches an extremely large NTE of *α*=−34 × 10^−6^ K^−1^ in Cd(CN)_2_ (ref. 7), where *α* is the coefficient of linear thermal expansion. In recent years, new materials in this category, such as ScF_3_ (ref. 8), have been identified.

Another avenue towards discovery of giant NTE is the utilization of a phase transition accompanied by large volume contraction on heating. The effectiveness of this approach was recognized widely by the giant NTE of the antiperovskite manganese nitrides Mn_3_*A*N, where *A* represents a metal or semiconducting element^9^, which use volume change caused by a magnetic transition (that is, magnetovolume effects). The giant NTE of Mn_3_*A*N strongly influenced subsequent NTE research, leading to the discovery of many phase-transition-type NTE materials such as La(Fe, Si, Co)_13_ (ref. 10) and MnCo_0.98_Cr_0.02_Ge (ref. 11), which use magnetovolume effects, SrCu_3_Fe_4_O_12_ (ref. 12) and Bi_0.95_La_0.05_NiO_3_ (ref. 13), which use intermetallic charge transfer, and 0.4PbTiO_3_–0.6BiFeO_3_ (ref. 14), which uses a ferroelectric transition.

One promising mother compound for NTE materials is layered ruthenate Ca_2_RuO_4_ (refs 15, 16, 17, 18, 19, 20, 21, 22). It undergoes a metal–insulator (MI) transition, which is apparently accompanied by volume expansion. Using dilatometry, we discovered giant NTE of large total volume change Δ*V*/*V*=6.7% and *α*=−115 × 10^−6^ K^−1^ (Δ*T* of ∼210 K) in reduced Ca_2_RuO_4_ (Supplementary Fig. 1). This gigantic volumetric NTE is too large to be attributable to the unit-cell volume NTE estimated by X-ray diffraction (XRD) study. We discuss microstructural effects that enhance the bulk NTE of sintered samples.

## Results

### Thermal-expansion properties

**Figure 1:**
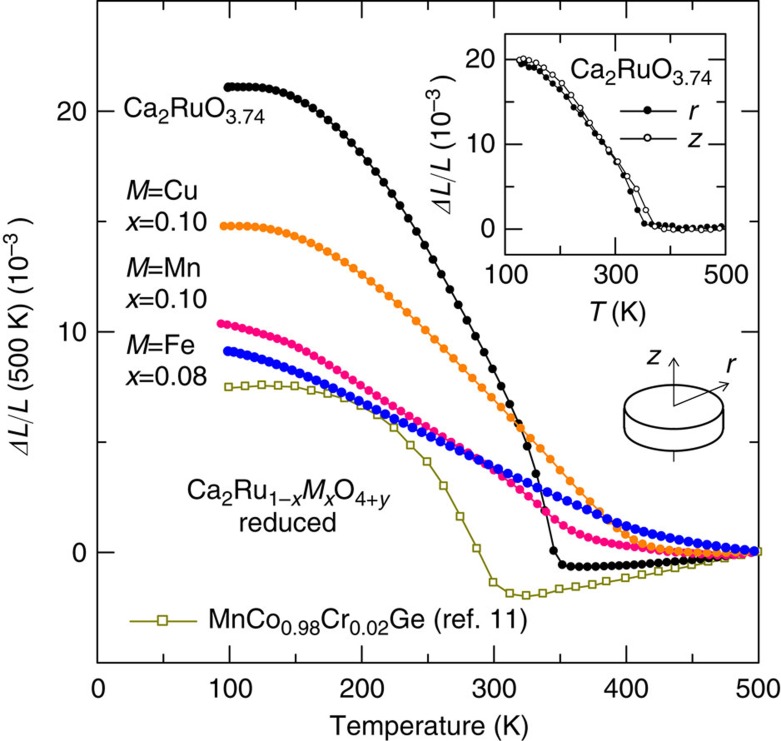
Linear thermal expansion Δ*L/L*of reduced layered ruthenates. The data were collected on a warming process using a laser-interference dilatometer (the *y* values are presented in Table 2). Reference temperature: 500 K. The reduced Ca_2_RuO_3.74_ exhibits giant negative thermal expansion (NTE) of *α*=−115 × 10^−6^ K^−1^ (*α*: coefficient of linear thermal expansion) over 200 K interval below 345 K. The vertical (*z*) and horizontal (*r*) expansions measured using a thermomechanical analyzer were found to be identical (inset). Therefore, this NTE is volumetric. The total volume change Δ*V*/*V* related to NTE reaches 6.7% at most. Substituting the transition-metal elements for Ru reduces the total volume change, but increases the onset of NTE. Particularly, the Fe-doped ruthenate exhibits temperature-linear behaviour below 500 K, which is favourable for practical applications. For comparison, data of MnCo_0.98_Cr_0.02_Ge^11^ are also shown.

First, the present NTE of Ca_2_RuO_4_ is compared with NTE that are presently known. Figure 1 shows linear thermal expansion Δ*L*(*T*)/*L* of Ca_2_RuO_4_ and Ca_2_Ru_1−*x*_*M*_*x*_O_4_ (*M*: Mn, Fe and Cu) in this study. The vertical (cylindrical, *z*) and horizontal (radial, *r*) expansions of the Ca_2_RuO_4_ sintered pellets are identical. Therefore, it is isotropic and volumetric NTE (inset of Fig. 1, see ‘Methods' section for conditions of linear thermal-expansion measurements). Parameters related to NTE for recently discovered giant NTE materials are presented in Table 1. The largest Δ*V*/*V* related to NTE reported until now is 3.2% for MnCo_0.98_Cr_0.02_Ge (ref. 11). Data of Δ*L/L* for this alloy are presented for comparison in Fig. 1. Data for Pu^23^ and YMn_2_ (ref. 24) are also included in Table 1 for comparison. These reference materials exhibit large volume contraction on heating at the phase transition, but they exhibit abrupt volume changes. Therefore, these materials are not categorized as NTE materials. Extreme volume contraction on heating of Pu is 5.4% in successive *δ*→*δ*′→*ɛ* phase transitions^23^. Δ*V*/*V* reaches 6.7% at most for the present Ca_2_RuO_4_. Such a large total volume change produces gigantic NTE of *α*=−115 × 10^−6 ^K^−1^ at *T*=135−345 K. The present Ca_2_RuO_4_ possesses greater volume contraction on heating at ambient pressure than all the listed materials. Moreover, it exhibits giant NTE over a wide *T* range, including room temperature.

Earlier studies have revealed that Ca_2_RuO_4_ undergoes a Mott MI phase transition from a high-*T* metallic to a low-*T* insulating state at *T*_MI_ of ∼360 K (refs 15, 16, 17). At this transition, the crystal structure also changes from the high-*T* L phase with a longer *c* axis to the low-*T* S phase with a shorter *c* axis while preserving the orthorhombic crystal structure of the *Pbca* symmetry. Some earlier studies have also found the appearance of NTE in Ca_2_RuO_4_ because the unit-cell volume *v* of the S phase is greater than that of the L phase. Detailed structural analysis^17^ has revealed volume contraction Δ*V*/*V* of ∼1% during heating from 100 to 400 K. Qi *et al*.^20^ reported successive phase transitions with Δ*V*/*V* of completely 0.9% of volume contraction on heating for Ca_2_Ru_0.933_Cr_0.067_O_4_ and NTE of *α*=−10 × 10^−6^ K^−1^ at *T*=120−400 K (Δ*V*/*V* of ∼0.8%) for Ca_2_Ru_0.90_Mn_0.10_O_4_ (ref. 21). The total volume change of the present Ca_2_RuO_4_, Δ*V*/*V*=6.7%, is much greater than those previous results.

Partial replacement of Ru by other elements alters the thermal-expansion properties of Ca_2_RuO_4_ such as the operating-temperature window Δ*T*, negative slope *α*, and the total volume change Δ*V*/*V*. We investigated the effects of three dopants, Mn, Fe and Cu (Fig. 1). The dopants Mn, Fe and Cu increase the onset of NTE, *T*_onset_, although they decrease the respective total volume changes: *T*_onset_=470 K and Δ*V*/*V*=3.1% for Ca_2_Ru_0.90_Mn_0.10_O_4_ (Mn0.10), *T*_onset_=500 K or higher and Δ*V*/*V*=2.8% for Ca_2_Ru_0.92_Fe_0.08_O_4_ (Fe0.08), and *T*_onset_=430 K and Δ*V*/*V*=4.4% for Ca_2_Ru_0.90_Cu_0.10_O_4_ (Cu0.10). Particularly, the Fe-doped ruthenate exhibits *T*-linear expansion in almost the entire range of *T* below 500 K, which is favourable for practical applications. Thermal expansion exhibits almost *T*-linear behaviour, even near the lowest temperature (95 K) used for the present dilatometry measurements. Therefore, NTE apparently continues down to the lower temperature. In that case, the total volume change Δ*V*/*V* might become greater than the present estimate of 2.8% (*T*=95–500 K).

### Effects of oxygen deficiency

**Figure 2:**
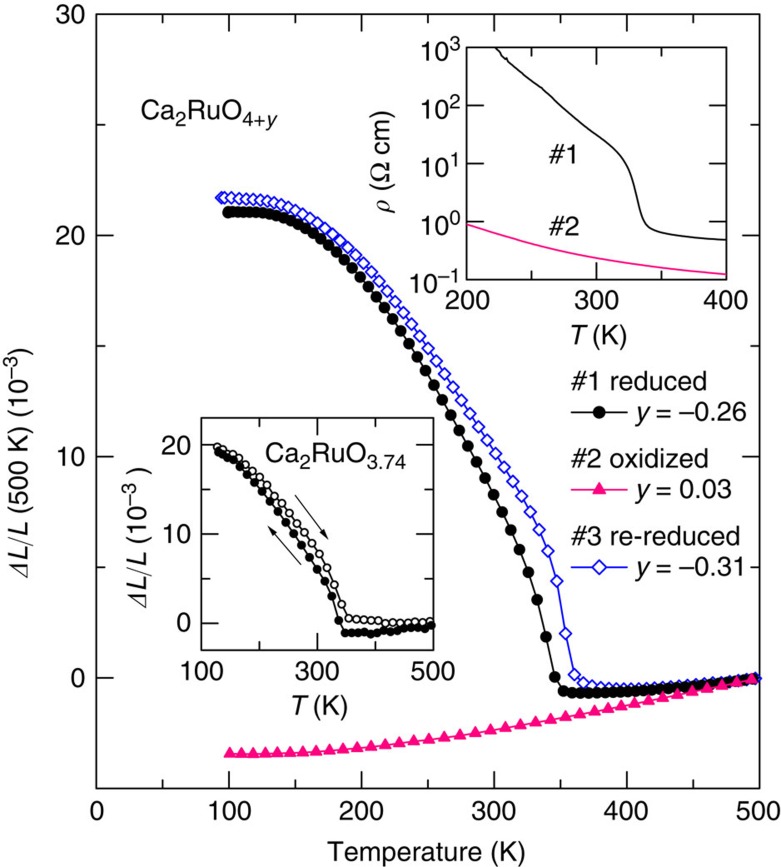
Linear thermal expansion Δ*L/L* of Ca_2_RuO_4+*y*_. The data were collected on a warming process using a laser-interference dilatometer. Reference temperature: 500 K. The hysteresis loop was observed in Δ*L/L* measurements using a thermomechanical analyzer for #1 (inset). The giant negative thermal expansion (NTE) of #1 is suppressed by the oxidizing procedure, but is fully recovered by the re-reducing procedure. Inset shows temperature dependence of resistivity *ρ*(*T*) for #1 and #2. The abrupt jump in *ρ*(*T*) at 345 K for #1 corresponds to the Mott metal-to-insulator transition, which coincides with the onset of NTE.

We can examine the differences between the present materials showing giant NTE and previous materials. Figure 2 shows linear thermal expansion Δ*L*(*T*)/*L* of reduced (#1), oxidized (#2), and re-reduced (#3) Ca_2_RuO_4_ in the present experiments (see ‘Methods' section for sample preparation conditions). The giant NTE of the reduced sample is suppressed dramatically by high-pressure oxidizing procedures. When this oxidized sample is reduced again, the giant NTE is recovered. The results presented above imply that differences in oxygen contents produce a striking difference in thermal-expansion properties. Evaluations of the oxygen contents by thermogravimetric analysis are *y*=−0.26(1), 0.03(1) and −0.31(1), respectively, for the reduced, oxidized and re-reduced samples in the notation of Ca_2_RuO_4+*y*_. Reports of an earlier study described that *y* fell within the range of −0.01(1) to +0.07(1)^15^. The present reduced ruthenates are regarded as having larger amounts of oxygen deficiency than the previous ones. Hereinafter, we use Ca_2_Ru_1−*x*_*M*_*x*_O_4+*y*_ notation as the present materials. The *y* values are presented in Table 2.

### High-temperature L to low-temperature S phase transition

The giant NTE of Ca_2_RuO_3.74_ seems to be triggered by the transition from the high-*T* metallic L phase to the low-*T* insulating S phase. The inset of Fig. 2 presents the temperature dependence of resistivity *ρ*(*T*) for the reduced and oxidized Ca_2_RuO_4+*y*_. The resistivity of the reduced sample indicates that the system undergoes the MI transition at *T*_MI_=345 K. The onset of NTE is almost identical to this MI transition. In contrast, resistivity of the oxidized sample shows no abrupt change that can be interpreted as prolonged high-*T* L phase down to lower temperatures. Corresponding to this MI transition, the anomaly appears in the magnetic susceptibility *χ*(*T*) (Supplementary Fig. 2). *χ*(*T*) is hysteretic (∼10 K). It therefore supports the first-order nature of this phase transition. Such hysteretic behaviour is confirmed also in the linear thermal expansion. The hysteresis loop was observed in dilatometry measurements (inset of Fig. 2). The onset of NTE is 340 and 355 K on cooling and warming processes, respectively. This loop behaviour is partly attributable to the first-order phase transition. The loop behaviour is reproducible through several successive measurements.

### Structural characterization of Ca_2_Ru_1−*x*_*M*_*x*_O_4+*y*_

**Figure 3:**
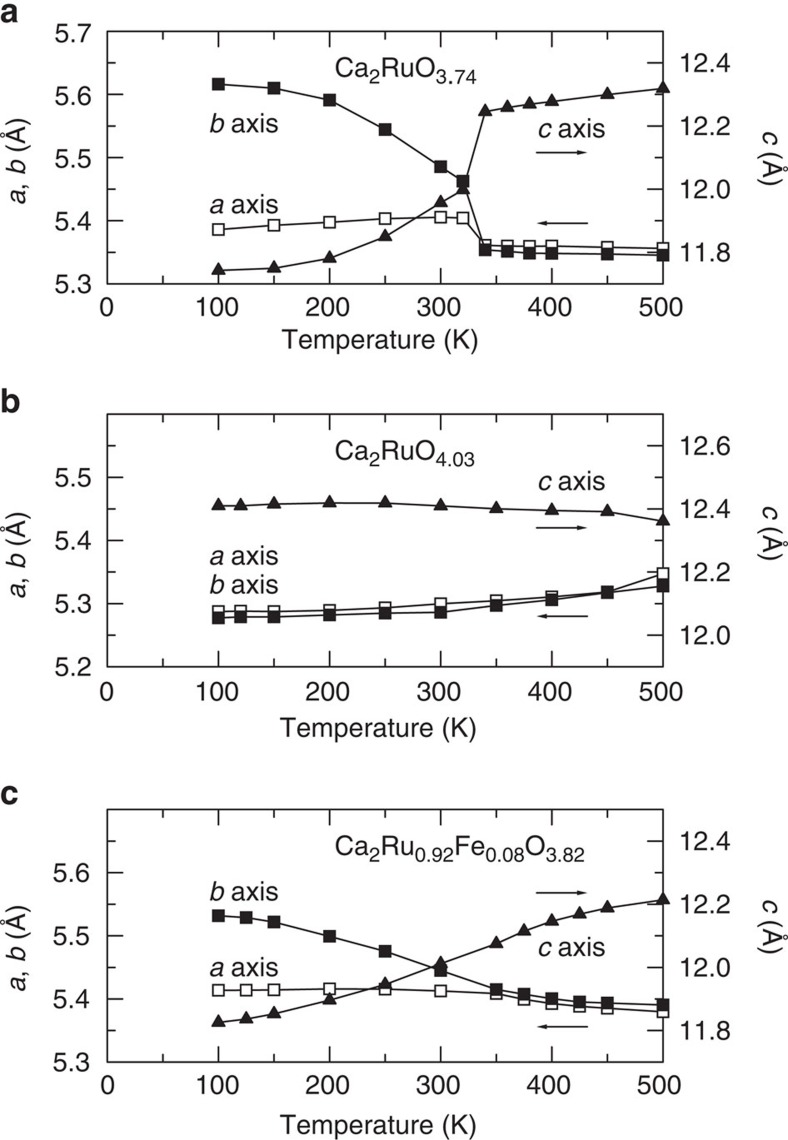
Temperature dependence of the lattice parameters. (**a**) Ca_2_RuO_3.74_, (**b**) Ca_2_RuO_4.03_ and (**c**) Ca_2_Ru_0.92_Fe_0.08_O_3.82_. These values are estimated based on Le Bail analysis of the XRD data. For Ca_2_RuO_3.74_, the *c* axis decreases abruptly below *T*_MI_=345 K. The S phase is characterized by highly anisotropic thermal expansion. The *a* and *b* axes increase, although the *c* axis decreases concomitantly with decreasing temperature. The present results confirmed that the negative thermal expansion appears in the S phase.

**Figure 4:**
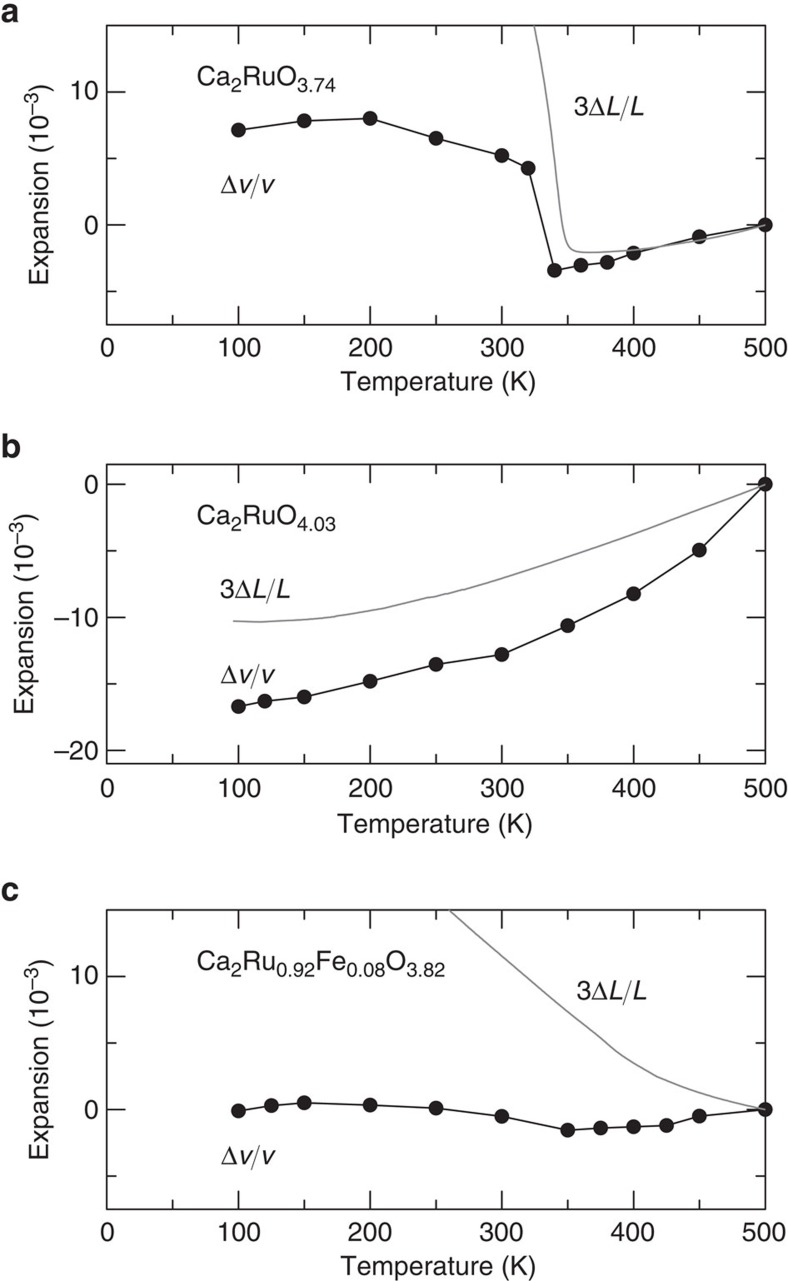
Volumetric thermal expansion of Ca_2_Ru_1−*x*_*M*_*x*_O_4+*y*_. (**a**) Ca_2_RuO_3.74_, (**b**) Ca_2_RuO_4.03_ and (**c**) Ca_2_Ru_0.92_Fe_0.08_O_3.82_. Results estimated from crystallographic (Δ*v*/*v*) measurements are compared with the dilatometry (3Δ*L*/*L*) results. Reference temperature: 500 K. The discrepancy is conspicuous when the dilatometric negative thermal expansion becomes large.

Giant NTE can be considered in terms of its crystal structure. The XRD profiles were refined using Le Bail method by RIETAN-FP^25^ (Supplementary Figs 3 and 4). The structural parameters such as the lattice constants (*a*, *b* and *c*) and the unit-cell volume (*v*) determined by the present refinements are presented in Table 2 and Figs 3 and 4. Although some differential peaks still exist for the statistics problem of data, the whole pattern fittings are accomplished fairly well, indicating that the peak positions are adequately refined and hence the obtained lattice parameters are reliable. The 111 peak (2*θ*∼24 deg.) is a single peak for both the L and S phases. The width of this peak is as narrow as those of other diffraction peaks such as the 002 peak (2*θ*∼14 deg.). In addition, the 200 and 020 peaks are split in both the L and S phases (insets of Supplementary Fig. 4). These two features support that the present crystals belong to orthorhombic symmetry. Through careful investigation of the extinction rule of *k*≠2*n* for 0*kl*, *l*≠2*n* for *h*0*l* and *h*≠2*n* for *hk*0, we conclude that the space group of the present crystals is *Pbca* for both the L and S phases. Note that the 200 and 020 peaks are still split in the high-*T* L phase, although the peak positions are quite close. The difference between the *a* axis and *b* axis parameters is significantly large compared with the standard deviations of the present analysis. These features indicate that, despite discontinuous change in lattice parameters, the space group is not changed across the transition. Because the difference between the *a* axis and *b* axis parameters is small, some ambiguity persists as to whether *a*>*b* or *a*<*b* for the L phase in the present analysis. Following a detailed neutron diffraction study^17^, we assumed the former.

Temperature-dependent XRD measurements provide additional information related to the mechanism behind the NTE. The S phase in which the giant NTE appears for Ca_2_RuO_3.74_ is characterized by highly anisotropic thermal expansion of lattice parameters: The *a* and *b* axes become longer although the *c* axis becomes shorter concomitantly with decreasing *T*. For Ca_2_RuO_3.74_, the *b* axis expands by 5.0% and the *c* axis contracts by 4.2% from 340 to 100 K. Consequently, the unit-cell volume *v* exhibits NTE behaviour. However, the dilatometric NTE of Δ*V*/*V*=6.7% is, surprisingly, too large to be ascribed to the unit-cell volume NTE, which is at largest 1%. For Ca_2_Ru_0.92_Fe_0.08_O_3.82_ (Fe0.08), which shows NTE in the entire *T* range below 500 K, the discontinuous change in lattice parameter related to the L-to-S phase transition is not clear, presumably because of Fe-doping effects. Although the *a* axis parameter becomes close to the *b* axis parameter above 350 K, the *c* axis parameter continues to increase gradually with *T* and is still remained in 12.2140(2) Å even at 500 K. As a result, Fe0.08 is also characterized by highly anisotropic thermal expansion of lattice parameters below 500 K: the *b* axis expands by 2.5% and the *c* axis contracts by 3.4% from 500 to 100 K. In this *T* range, the sintered body exhibits the NTE, though the unit-cell volume exhibits nearly zero thermal expansion. In contrast, for Ca_2_RuO_4.03_, which shows weak or no NTE, the unit-cell volume variation in *T* is close to the result obtained using dilatometry (3Δ*L/L*). In the prolonged L phase, the lattice parameters show no drastic *T* dependence, contrary to Ca_2_RuO_3.74_.

## Discussion

Some materials are reported to have dilatometric NTE greater than the crystallographic unit-cell volume NTE in sintered ceramics^1^. One well known example is β-eucryptite^26^,^27^, which has been widely used as a practical thermal-expansion compensator. On cooling from 1,073 to 293 K, the *a* axis in the hexagonal unit cell contracts by 0.62%, whereas the *c* axis expands by 1.39%, which yields net unit-cell volume NTE of 0.15%. In contrast, the dilatometric NTE observed in sintered β-eucryptite reaches 1.7%, which is greater than 10 times of the crystallographic NTE (Table 3). Such a remarkable enhancement of thermal expansion has been ascribed to the characteristic microstructure of the sintered body, and particularly to spontaneous microcracking that originates from extremely anisotropic thermal expansion. Similar effects should be considered for Ca_2_RuO_4+*y*_ because it also exhibits extremely large anisotropic thermal expansion in the lattice parameters when the giant NTE appears.

**Figure 5:**
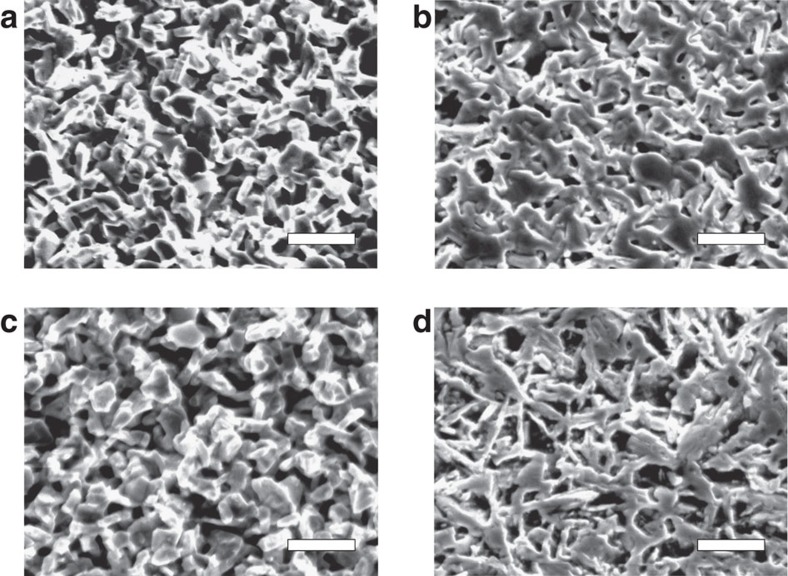
Microscopic images by scanning electron microscopy. (**a**) Reduced Ca_2_RuO_3.74_ (#1), (**b**) oxidized Ca_2_RuO_4.03_ (#2), (**c**) re-reduced Ca_2_RuO_3.69_ (#3) and (**d**) reduced Ca_2_Ru_0.92_Fe_0.08_O_3.82_. The morphology is apparently reversible in successive heat treatment: #1→#2→#3. Scale bars, 20 μm.

However, the NTE of the reduced ruthenates is not accompanied by pronounced hysteresis: a characteristic of microcracking. Scanning electron microscope (SEM) observations do not confirm microcracking, but confirmed voids (Fig. 5). The preliminary measurements of specific gravity using Archimedes' method suggest that the density of the present ceramic samples is 80–90%. Compared with materials such as β-eucryptite and MgTi_2_O_5_ (ref. 28), for which microcracking effects have been considered, the onset temperature of the anisotropic thermal expansion is much lower (around room temperature). Therefore, cumulated inner stresses are expected to be much smaller for the reduced ruthenates. Even without generating microcracks, a porous sintered body might exhibit bulk NTE if extremely anisotropic thermal expansion of the grains produces deformation, consuming open spaces (voids) on heating. More detailed micrographic observations must be done to clarify the microstructural effects.

Here, we summarize the effects of oxygen deficiency on the NTE. Thermal-expansion properties of Ca_2_RuO_4_ show some scattering among results reported by different research groups. For example, Qi *et al*.^20^ reported no NTE of the unit-cell volume *v* in pure Ca_2_RuO_4_, although Braden *et al*.^15^ reported NTE of Δ*v*/*v*=1%. The discrepancy might be explained by differences in oxygen contents. The effects of oxygen deficiency might become remarkable for the doped ruthenates. The present Fe-doped ruthenate exhibits almost constant unit-cell volume, whereas the sample of Qi *et al*.^21^ preserved NTE of Δ*v*/*v*=0.8%. The SEM observation (Fig. 5) shows that the morphology changes from #1 to #2 despite heat treatment at much lower temperatures (773–823 K) than the sintering temperature (1,573–1,623 K). In addition, thermal expansion of a sintered body consisting of grains with highly anisotropic thermal expansion and voids might depend on the elastic properties of the grains. The NTE behaviour of the ruthenates is sensitive to the oxygen contents. The oxygen deficiency might affect the bulk thermal expansion via the alternation of elastic properties and morphology as well as intrinsic crystallographic parameters. Further structural analyses are expected to be useful to elucidate the roles of oxygen deficiency.

This study provides a strategy for realizing giant NTE, elaboration of sintered-body structure by combining grains having extremely anisotropic thermal expansion and moderate amount of voids. Control of thermal expansion using specific characteristics of materials, particularly in a negative *α* region, is highly limited in an operating temperature and/or a magnitude of *α* because of the severe constraint of available materials. To overcome these difficulties, designated structures consisting of two materials having different (positive) thermal expansions and voids are proposed as an artificial material showing NTE^29^,^30^. The present result is expected to stimulate those activities as a ‘natural' counterpart of the artificial structures. The capability of thermal-expansion compensation is related directly to dilatometric NTE measured in a finite size of materials, not necessarily to unit-cell volume thermal expansion, as might be apparent in the case of β-eucryptite. A high-performance thermal-expansion compensator will be realized by optimizing the microstructure of a material, such as amount of voids, micrograin orientation, and binding state of grains, as well as intrinsic characteristics of materials such as anisotropic thermal expansion and elastic properties.

## Methods

### Sample fabrication

Sintered polycrystalline samples of Ca_2_Ru_1−*x*_*M*_*x*_O_4_ (*M*=Mn, Fe and Cu) were prepared using solid-state reactions. Powders of CaCO_3_, RuO_2_, Mn_3_O_4_, Fe_3_O_4_ and CuO (purity: 99.9% or higher) weighed at appropriate molar ratios were mixed and heated in air at 1,273–1,373 K for 18 h. The obtained powder was reground, pressed into a pellet and sintered under flowing mixed gas of O_2_ 0.02 MPa/Ar 0.08 MPa at 1,573–1,623 K for 48 h. Materials of this type are designated as ‘reduced' samples. These reduced samples were heated under 0.5 MPa of O_2_ at 773–823 K for 50 h. Materials of this type are designated as ‘oxidized' samples. Then, the oxidized samples were heated under flowing mixed gas of O_2_ 0.02 MPa/Ar 0.08 MPa at 1,573–1,623 K for 48 h. Materials of this type are designated as ‘re-reduced' samples. We analysed the chemical compositions of the samples using energy dispersive X-ray spectroscopy (EDX, Genesis2000K; EDAX). The oxygen contents were investigated using thermogravimetric analysis (TGA, TG-DTA2020SAH; Bruker Analytik GmbH). XRD was used to identify the samples as layered ruthenate Ca_2_Ru_1−*x*_*M*_*x*_O_4_ and to analyse their crystal structure. The XRD analyses were conducted at 295 K (RINT2100; Rigaku) and at 100–500 K (D8 Advance; Bruker Analytik GmbH) with Cu K*α* radiation. The sample surfaces were observed using a scanning electron microscope (SEM, VE-7800; Keyence). Specific gravity was measured by an Archimedes' method (GR-200 and AD-1653; A&D).

### Characterization of thermal dependence of sample properties

Linear thermal expansion Δ*L*(*T*)/*L* along with the horizontal direction was measured using a laser-interference dilatometer (LIX-2; Ulvac) with a warming process. Δ*L*(*T*)/*L* along with the vertical and the horizontal directions on both warming and cooling processes were measured using a thermomechanical analyzer (TMA8310; Rigaku). For sintered polycrystalline samples, Δ*L/L* is related directly to the volume (*V*) expansion in a manner of Δ*L/L*=(1/3)Δ*V*/*V*. Temperature-dependent resistivity *ρ*(*T*) was measured using a conventional four-probe method (PPMS: Quantum Design). Temperature-dependent magnetization *M*(*T*) was measured at 1T using a superconducting quantum interference device magnetometer (MPMS; Quantum Design).

### Data availability

The data that support the findings of this study are available from the corresponding author on request.

## Additional information

**How to cite this article:** Takenaka, K. *et al*. Colossal negative thermal expansion in reduced layered ruthenate. *Nat. Commun.* **8,** 14102 doi: 10.1038/ncomms14102 (2017).

**Publisher's note**: Springer Nature remains neutral with regard to jurisdictional claims in published maps and institutional affiliations.

## Supplementary Material

Supplementary Information Supplementary Figures

## Figures and Tables

**Table 1:** Parameters related to negative thermal expansion for recently discovered giant negative thermal-expansion materials.

	**Δ*****V*****/*****V*** **(%)**	***T***_**NTE**_ **(K)**	**Δ*****T*** **(K)**	***α*** **(10**^**−6**^ **K**^**−1**^)*	**Structure**†	**Method**‡	**Reference**
ZrW_2_O_8_	1.2	<425	425	−9	Cubic	D/N	6
Cd(CN)_2_	2.1	170–375	205	−34	Cubic	X	7
Mn_3_Ga_0.7_Ge_0.3_N_0.88_C_0.12_	0.5	197–319	122	−18	Cubic	D	9
LaFe_10.5_Co_1.0_Si_1.5_	1.1	240–350	110	−26	Cubic	D	10
MnCo_0.98_Cr_0.02_Ge	3.2	122–332	210	−52	Orthorhombic	D	11
SrCu_3_Fe_4_O_12_	0.4	180–250	70	−20	Cubic	X	12
Bi_0.95_La_0.05_NiO_3_	2.0	320–380	60	−82	Triclinic	D	13
0.4PbTiO_3_–0.6BiFeO_3_	2.7	298–923	625	−13	Tetragonal	X	14
Pu	5.4	337–480§	—	—	Cubic/Tetragonal	D	23
YMn_2_	4.7	75||	—	—	Cubic	X	24

Parameters of materials with phase transition accompanied by large volume contraction on heating, not broadened, are also listed for comparison.

^*^Averaged value when the material is anisotropic.

^†^For NTE region or lower temperature, larger-volume phase.

^‡^D, dilatometry; N, neutron diffraction; X, X-ray diffraction.

^§^Successive transitions.

^||^In warming process.

**Table 2:** Crystallographic parameters of layered ruthenates obtained from Le Bail analysis of the X-ray diffraction data at 295 K.

	**Ca**_**2**_**RuO**_**4+*****y***_	**Ca**_**2**_**RuO**_**4+*****y***_	**Ca**_**2**_**RuO**_**4+*****y***_	**Mn0.10**	**Fe0.08**	**Cu0.10**
	**Reduced**	**Oxidized**	**Re-reduced**	**Reduced**	**Reduced**	**Reduced**
	***y*****=−0.26 (1)**	***y*****=0.03 (1)**	***y*****=−0.31 (1)**	***y*****=−0.27 (2)**	***y*****=−0.18 (2)**	***y*****=−0.18 (1)**
*a* (Å)	5.4102 (3)	5.3295 (3)	5.4015 (2)	5.3813 (3)	5.4156 (2)	5.4193 (2)
*b* (Å)	5.4926 (4)	5.3089 (3)	5.4790 (3)	5.3973 (3)	5.4481 (3)	5.4860 (4)
*c* (Å)	11.9511 (3)	12.4505 (6)	11.9438 (4)	12.0926 (7)	12.0012 (4)	11.9717 (5)
*b*/*a*	1.0152 (2)	0.9961 (2)	1.0143 (2)	1.0030 (2)	1.0060 (2)	1.0123 (2)
*c*/*a*	2.2090 (2)	2.3361 (3)	2.2112 (2)	2.2472 (4)	2.2160 (2)	2.2091 (2)
*v* (Å^3^)	355.14 (6)	352.27 (6)	353.47 (5)	351.22 (7)	354.09 (5)	355.92 (6)

**Table 3:** Parameters related to negative thermal expansion for materials with highly anisotropic structural distortion.

	**Δ*****V*****/*****V*** (%)*	***T***_**NTE**_ **(K)**	**Δ*****T*** **(K)**	***α*** **(10**^**−6**^**K**^**−1**^**)**†	**Structure**‡	**Method**§	**Reference**
β-eucryptite	0.15	293–1,073	780	−0.6	Tetragonal	X	26
	1.7			−7.6		D	
Ca_2_RuO_3.74_	1.0	135–345	210	−17	Orthorhombic	X	This work
	6.7			−115		D	
Ca_2_Ru_0.92_Fe_0.08_O_3.82_	0||	100–500	400	+2.5	Orthorhombic	X	This work
	2.8			−28		D	

^*^For dilatometry, it is estimated using the relation of Δ*L/L*=(1/3)Δ*V*/*V.*

^†^Averaged value when the material is anisotropic.

^‡^For NTE region.

^§^D, dilatometry; X, X-ray diffraction.

^||^Crystallographic volumetric thermal expansion is nearly zero.
