# Supplementary material for: Colossal negative thermal expansion in reduced layered ruthenate
Source: Nat Commun. 2017 Jan 10;8:14102. doi: 10.1038/ncomms14102 (PMC5234094; doi:10.1038/ncomms14102)
Supplement: Supplementary Information — Supplementary Figures [file ncomms14102-s1.pdf]

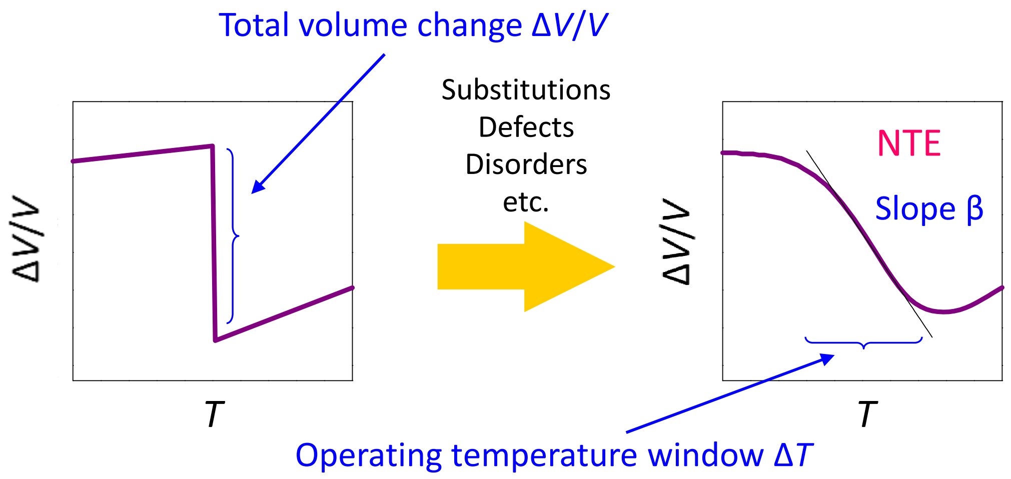

Supplementary Figure 1 | Concept of total volume change $\Delta V/V$ related to negative thermal expansion (NTE). In the case of phase-transition-type NTE materials, "slope" β (coefficient of *volumetric* thermal expansion) shares a tradeoff relation with the width of the operating-temperature window ΔT, roughly described as $\Delta V/V$~$|\beta|\Delta T$. For an isotropic material, a coefficient of *linear* thermal expansion α is related to β as $\beta=3\alpha$. A sintered body can be treated as isotropic because its linear thermal expansion is equivalent to the average of expansions in the three crystallographic directions (see *Methods* for characterization of thermal dependence of sample properties). Therefore, coefficients α and β are not intrinsic for such phase-transition-type materials. Instead, the total volume change $\Delta V/V$ is the intrinsic index indicating the potential of NTE.

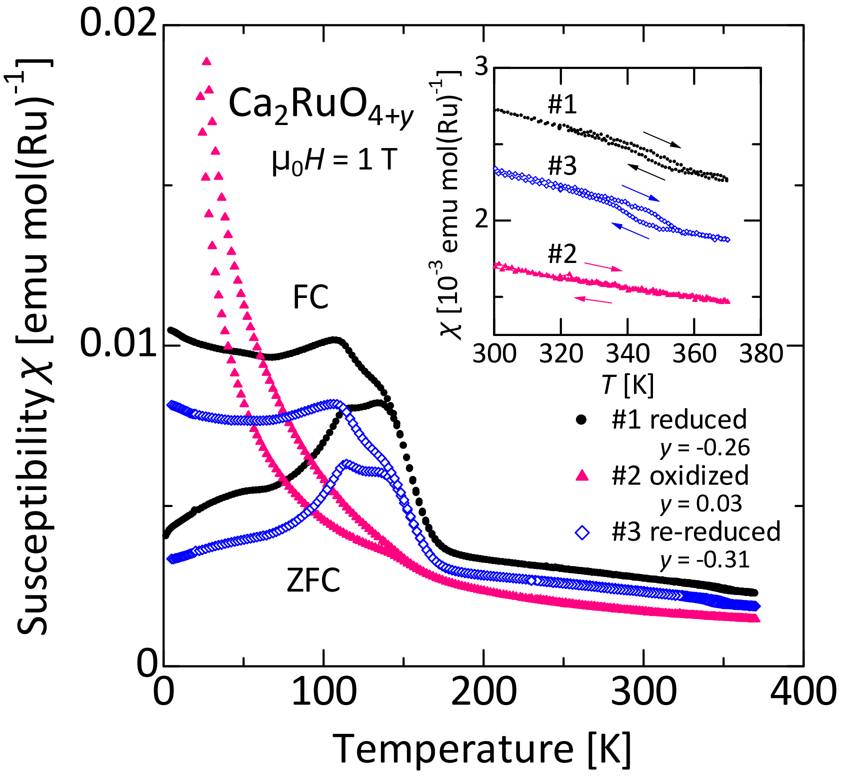

Supplementary Figure 2 | Temperature dependence of magnetic susceptibility χ(*T*) of Ca_2RuO_{4+y}. The applied field $\mu_0 H$ is 1 T. "ZFC" and "FC" respectively represent the zero-field-cooled and field-cooled data. The susceptibility of the reduced sample resembles data collected for the S-phase Ca_2RuO_4 [15], indicating that the antiferromagnetic order is realized below 110 K. Such magnetic behavior is reproduced for the re-reduced sample. Inset: The magnified susceptibility near the phase transition shows hysteresis related to the metal-insulator transition for the reduced and re-reduced samples.

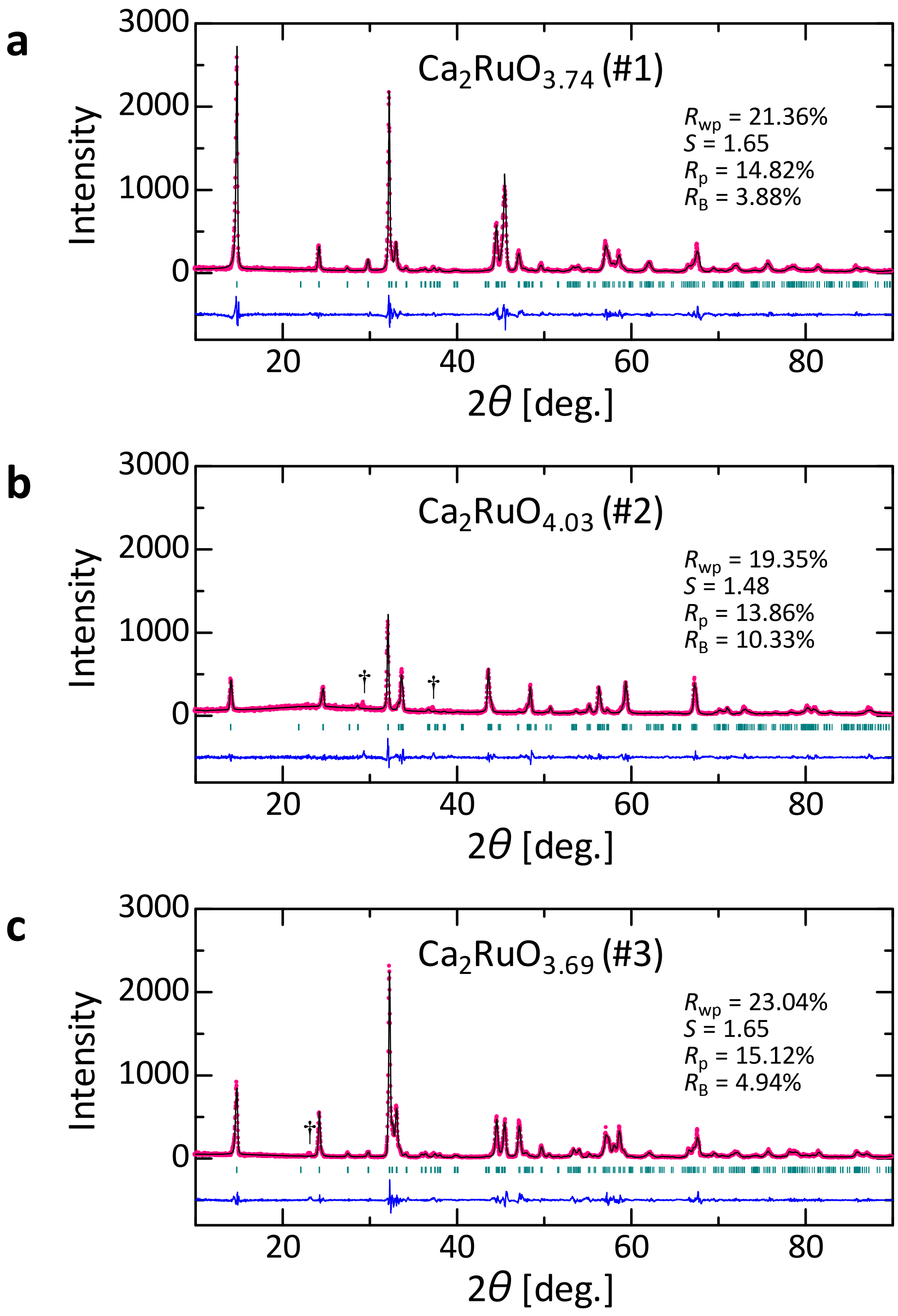

Supplementary Figure 3 | Le Bail refinement of Ca_2RuO_{4+y}. The experimental (solid circle) and fitted (line) X-ray diffraction pattern at room temperature (295 K) are shown. Vertical bars under the diffraction peaks show the Bragg reflection positions. The plots under the bars represent residues. The crystallographic parameters calculated from these refinements are presented in Table 2. All of the observed peaks except a few weak peaks of unknown cause (indicated by a dagger †) can be indexed based on the orthorhombic *Pbca* symmetry. The extinction rule predicts that the 011 and 101 peaks (~ 18 deg.) and the 110 peak (~ 23 deg.), for example, are missing in *Pbca* symmetry, which is consistent with the present data. The diffuse feature at 25 deg. in the middle panel is an artificial structure caused by diffuse scattering from the glass substrate.

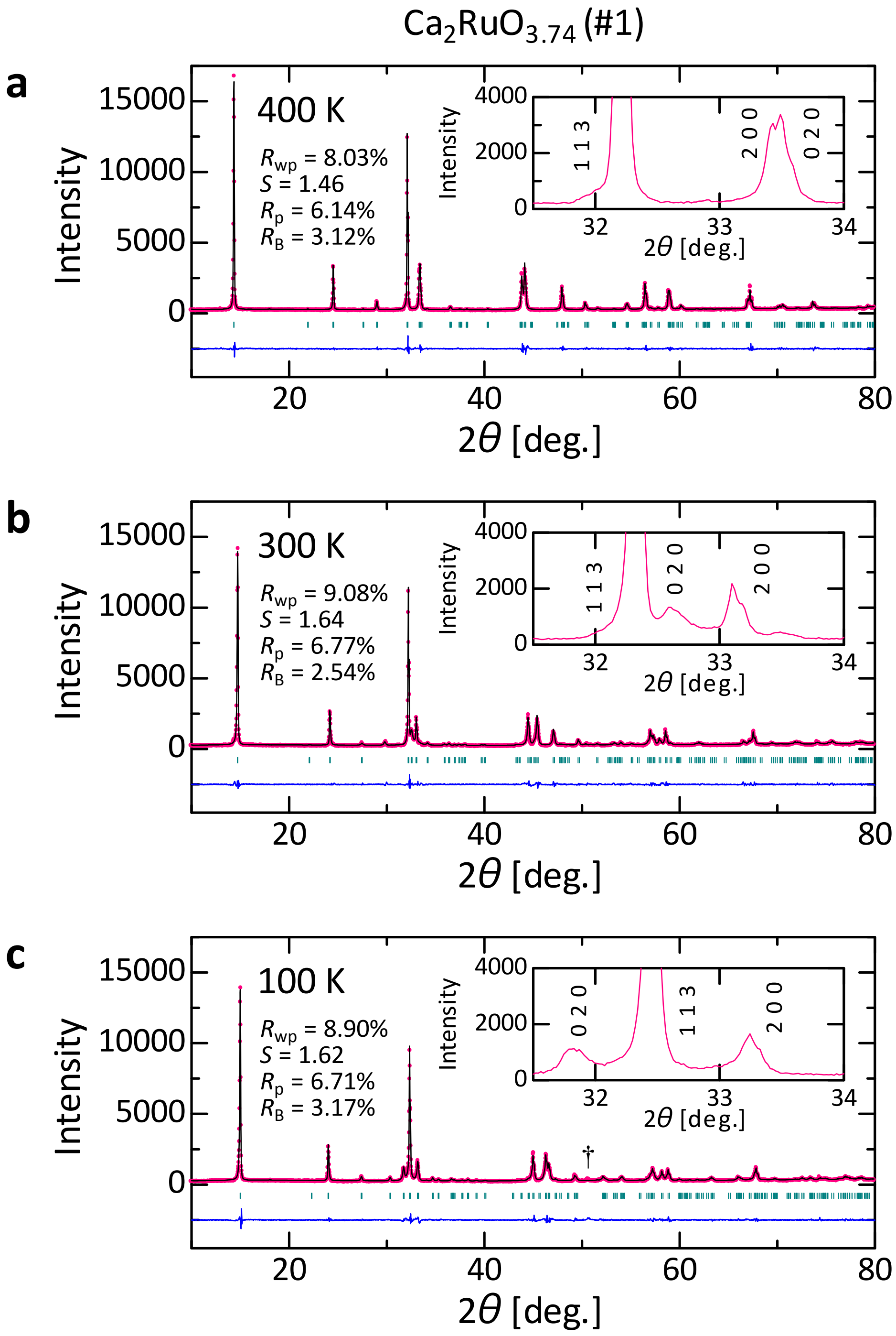

Supplementary Figure 4 | Le Bail refinement of $Ca_2RuO_{3.74}$. The experimental (solid circle) and fitted (line) X-ray diffraction pattern at 100 K, 300 K, and 400 K are shown. Vertical bars under the diffraction peaks show the Bragg reflection positions. The plots under the bars represent residues. The crystallographic parameters calculated from these refinements are presented in Figs. 3 and 4. All of the observed peaks except a weak peaks of unknown cause (indicated by a dagger †) can be indexed based on the orthorhombic *Pbca* symmetry. Insets show the magnified X-ray diffraction profiles around 33 deg. The 200 and 020 peaks are still split in the high-*T* L phase, indicating that the space group is unchanged across the transition.
